# Bridging data management platforms and visualization tools to enable ad-hoc and smart analytics in life sciences

**DOI:** 10.1515/jib-2022-0031

**Published:** 2022-09-08

**Authors:** Christian Panse, Christian Trachsel, Can Türker

**Affiliations:** Functional Genomics Center Zurich (FGCZ), University of Zurich/ETH Zurich, Winterthurerstrasse 190, CH-8057 Zurich, Switzerland

**Keywords:** accessible, findable, interoperable and reusable (FAIR), integrations for data analysis, open research data (ORD), workflow

## Abstract

Core facilities have to offer technologies that best serve the needs of their users and provide them a competitive advantage in research. They have to set up and maintain instruments in the range of ten to a hundred, which produce large amounts of data and serve thousands of active projects and customers. Particular emphasis has to be given to the reproducibility of the results. More and more, the entire process from building the research hypothesis, conducting the experiments, doing the measurements, through the data explorations and analysis is solely driven by very few experts in various scientific fields. Still, the ability to perform the entire data exploration in real-time on a personal computer is often hampered by the heterogeneity of software, the data structure formats of the output, and the enormous data sizes. These impact the design and architecture of the implemented software stack. At the Functional Genomics Center Zurich (FGCZ), a joint state-of-the-art research and training facility of ETH Zurich and the University of Zurich, we have developed the B-Fabric system, which has served for more than a decade, an entire life sciences community with fundamental data science support. In this paper, we sketch how such a system can be used to glue together data (including metadata), computing infrastructures (clusters and clouds), and visualization software to support instant data exploration and visual analysis. We illustrate our in-daily life implemented approach using visualization applications of mass spectrometry data.

## 1 Introduction

**Figure 1:**
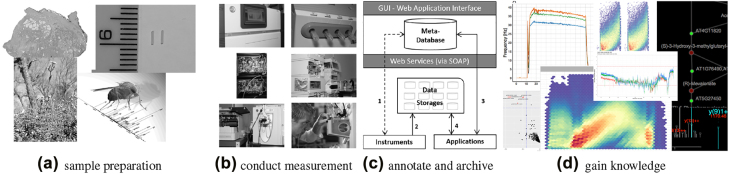
Sketch of a typical omics workflow.

Core facilities aim at supporting scientific research where routinely terabytes of archivable raw data are produced every year. This data is annotated, pre-processed [1], quality-controlled [2], and analyzed, ideally within pipelines. Additionally, reports are generated and charges are sent to the customers to conclude the entire support process. Typically, data acquisition, management, and analysis cycles are conducted in parallel for multiple projects with different research questions, involving several experts and scientists from different fields, such as biology, medicine, chemistry, statistics and computer science. Figure 1 sketches a typical omics workflow: (a) sample preparation of different organisms and tissues (human – a fresh frozen tissue with indicated cancer and benign prostate tissue areas, Arabidopsis/*Drosophila melanogaster* [3] model organism), (b) sequencing, and mass spectrometry are typical methods of choice to perform measurement based on the prepared samples in the *omics* fields, (c) handling and archiving data well as metadata with special emphasis on the reproducibility of the measurement results, (d) ad-hoc application of largely known community developed bioinformatics tools and visualization techniques, exploiting not only the raw data but also the associated metadata.

**Figure 2:**
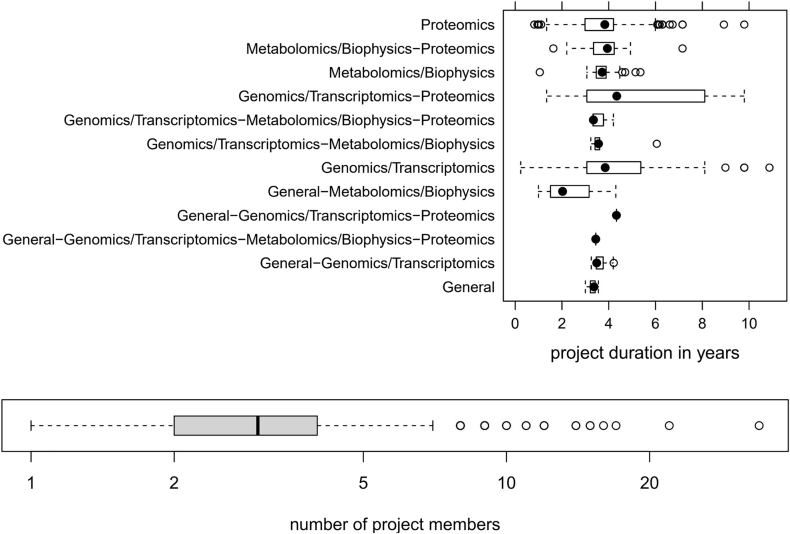
Project duration of different omics areas and number of project members.

It is important to note that most of the omics projects run over several years. Figure 2 depicts the typical duration of omics projects and the involved number of project members, as we have witnessed in our core facility over almost two decades.

The interdisciplinary nature of such scientific projects requires a powerful and flexible platform for data annotation, communication, and data exploration. Information visualization techniques [4, 5], especially interactive ones, are well suited to support the exploration phase of the conducted research. However, general support for a wide area of applications is often hampered for different reasons:

– Heterogeneity of structure and missing implementation of standards disable easy bundling of existing applications.
– Prototype applications are not deployable and maintainable in a more extensive system setup as it is needed for production use.
– Initial costs per user are high [ [6], section 4.3].
– Scientists usually have limited programming skills.

**Figure 3:**
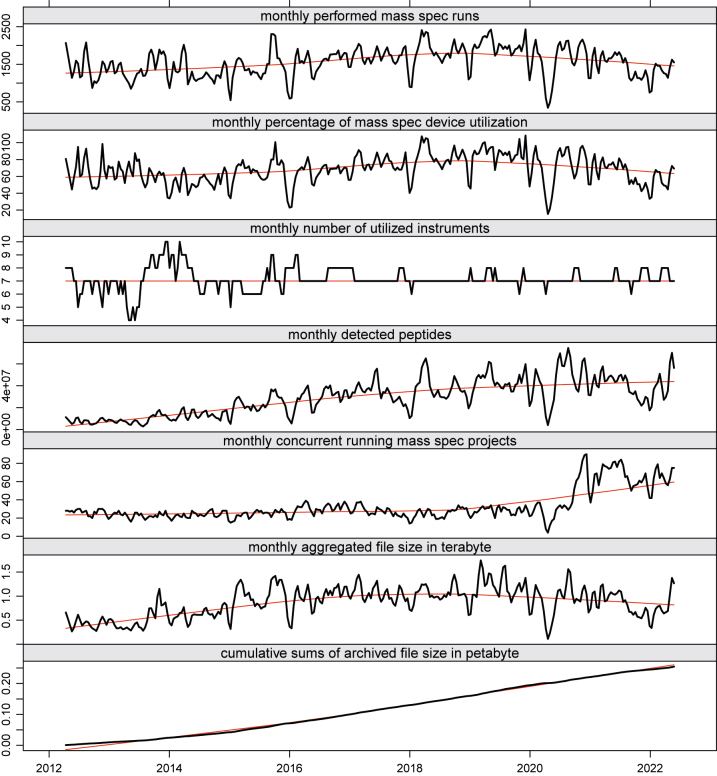
Key performance indicators.

The graphs in Figure 3 depict some *key performance indicator trends* while plotting a monthly sliding window over the aggregated data of conducted runs, the number of concurrent running research projects, and the number of detected peptides (proteins) of our mass spectrometry unit.

Throughout the years, we observed the phenomenon that traditional core IT development cannot cope with the pace of the evolution cycles of data analysis applications [1, 7]. Sometimes, new data analysis tools become obsolete before being fully integrated into the core IT system. Consequently, the core IT development team needs to carefully evaluate the latest trends in data analysis and assess which ones should be implemented first, considering not only scientific but also economic factors. At our core facility, we are faced with fundamental data processing and analysis problems due to challenging data formats used in the different scientific areas. In the following, we provide a brief example on a default proteomics workflow, as sketched in Figure 1, but note that the system analogously serves for other technology areas at our facility, such as *sequencing, genomics, metabolomics* and *single cell analysis*.

In the following, we will use several acronyms, which we listed in Table 1.

**Table 1:** List of used biochemistry acronyms.

ADP	Adenosine diphosphate
LC	Liquid chromatography
LTQ	Linear ion trap MS
MS	Mass spectrometry
MS2	Two or more mass spectrometers are coupled together
m/Z	Mass–ion charge ratio
STY	Amino acids 1-letter codes for Serine, Threonine, and Tyrosine
TIC	Total ion count
PTM	Post translational modification
QC	Quality control

**Use case**. In mass spectrometry, for instance, the mass charge ratio of ions is determined and the data is recorded as a spectrum consisting of a mass to charge (m/Z) axis (*x* coordinates) and an intensity axis (y coordinates). In proteomics – *“Everything in life is driven by proteins.”* [8] – the ions of interest are generated from peptides separated by liquid chromatography in time (30 min up to several hours) and recorded with high scan speed (up to 40 Hz). These quickly result in datasets of thousands of scans, each containing several hundreds of ions. Additional complexity is added to the data by the fact that the spectra can be recorded on different mass spectrometer (MS) levels. On MS level 1 (MS1), a simple survey is performed, while on MS level 2 (MS2), a specific ion from an MS1 scan is selected, isolated, fragmented, and the resulting fragment ions are recorded. On MS level 3 (MS3), an ion from an MS2 scan is selected for further fragmentation. To make matters more complicated, on MS2 and higher, different types of activation techniques can be selected for fragmentation. This setup allows generating complex experiments which result in detailed nested data. To answer a scientific question with such mass spectrometry data, it is necessary to filter out some selected ions of interest from several million ions distributed over the data structure. Some typical questions in this area are:

– Which proteins can be identified in an organism under which condition?
– Can particular post-translational modifications (PTMs), e.g., Phosphorylation on STY, be detected or new ones discovered?
– How do protein abundance levels or PTM patterns change under different conditions?

Ideally, data is measured, automatically transferred to the storage, and fed into a meta-database using automated robots in a large-scale high-throughput manner by using proprietary software provided by the vendors or free software packages. The analysis part is often implemented by using environments such as The Comprehensive R Archive Network (CRAN) [9] and Bioconductor [10–12], and packages like shiny [13], allowing to integrate of largely known bioinformatics tools, e.g., [14–19], and visualization techniques [4, 5, 10, 20], [21], [22]. The packages knitr [23] and rmarkdown [24] facilitate automated and reproducible report generation. The art is in bring all these tools together to document the research steps so such that the data involved can be provided and interpreted correctly. For that, an integrative platform is needed.

**Contributions**. It is essential to note this paper focuses neither on presenting a new visualization tool nor on introducing a new integrative platform. The goal is to share experiences we made in quickly developing visualization applications in the area of Life Sciences based on an integrative platform on which we have successfully implemented and run for more than ten years at our core facility. We will demonstrate how to couple and run real visualization applications on our generic integrative platform, called B-Fabric. As an example, we have chosen the visualization of mass spectrometry data. Here, we will show how an existing software (an R package, e.g., [25]) can be integrated into a shiny framework. The B-Fabric platform will only be sketched at a level needed to understand this paper’s message. We refer to [7, 26, 27] for more details on B-Fabric. In the remainder of this paper, we will show how to link applications to B-Fabric to detect a given set of mass patterns by applying the full bandwidth of information visualization techniques developed in the last thirty years and available through environments like R shiny [13]. While this particular application focuses on identifying post-translational modification (PTM) signatures [25, 28], the application itself could be exchanged easily by others. Also, traditional visualization systems, such as Spotfire [29], can be used on the higher layer of our architecture.

The paper organization is as follows. This section tried to motivate the need for our proposed solution by using the method of choice for measuring proteomics-related data. Section 2 provides a brief overview of the data management system, while Section 3 presents two typical application examples, one interactive and one with a graphical user interface. The final section provides some *lessons learned* and concludes with the described solution.

## 2 Integrative system platform

Core facilities require a data management and processing platform to cope with the massive data and query sizes of today’s research, as well as with immense dynamics of evolving analysis methods and tools that can emerge and disappear at a barely perceptible pace. A successful solution has to separate the different concerns levels and avoid bottlenecks caused by a limited number of system programmers and data analysis experts, as well as by restricted storage and computing resources. Concerning data management, it is essential to split the meta data from the actual data content.

**Figure 4:**
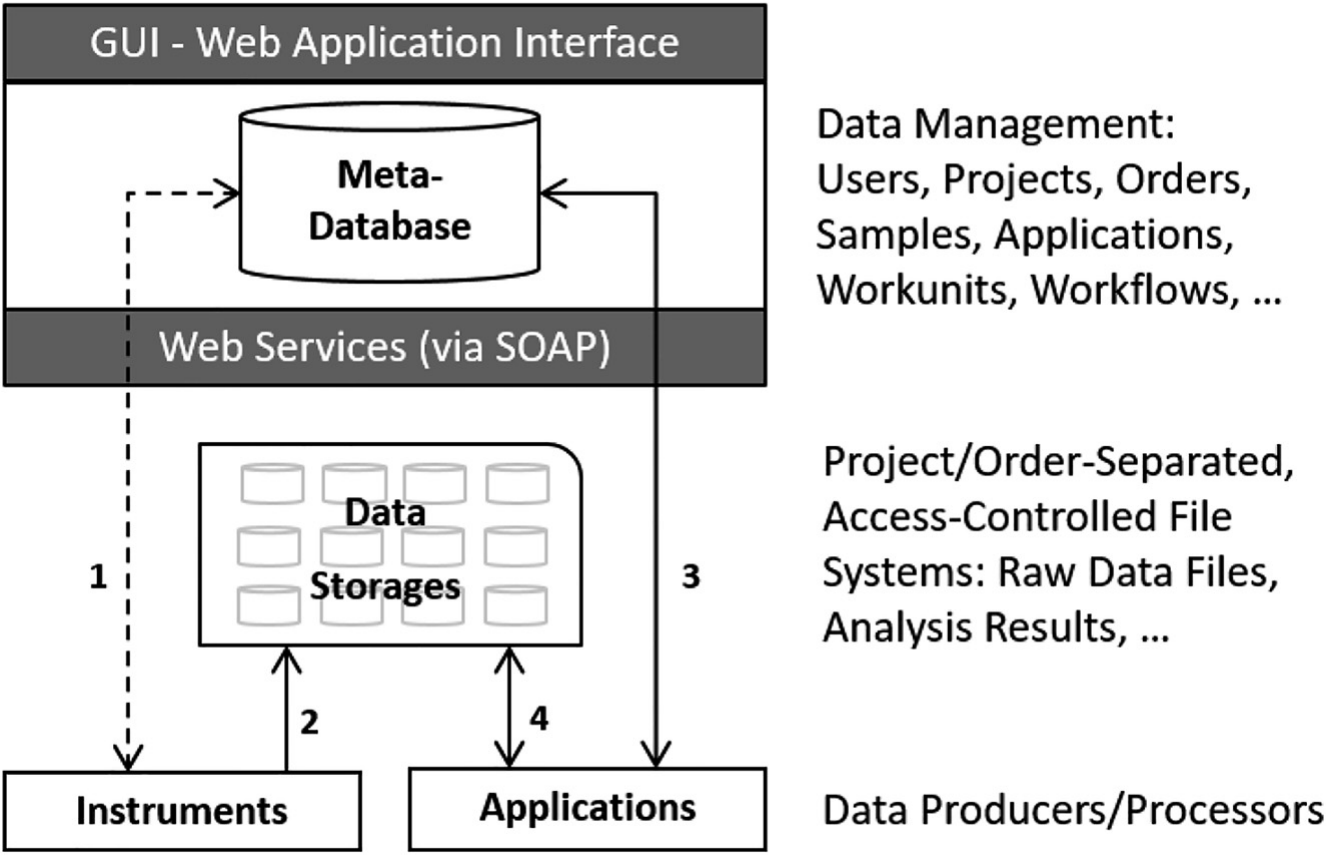
Rough sketch of system architecture.

Typical system solutions for Life Sciences research only focus on specific tasks. For instance, analysis tools such as TIBCO Spotfire [29] or SBEAMS [30], myProMS [31], or PeakForest [32] support data preparation and discovery features that help to shape, enrich, and transform Life Sciences data. Analytics platforms such aus KNIME [33], TeraData [34], or GenomeSpace [15] concentrate on enabling users to leverage and pipeline their preferred tools across multiple data types. Tools like the CLC Genomics Workbench [35], PanoramaWeb [36], or Google Genomics mainly provide technologies to analyze, visualize, and share *life sciences* data. Systems like Seven Bridges [37] go a step further by including basic project and sample management features to organize and share imported and processed data in a more fine-grained, access-controlled fashion. However, to the best of our knowledge, there is no single software that supports the entire needs of a core facility, namely the *administrative* tasks such managing all required information about the users (researchers), projects, orders, computers, instruments, storages, services, instrument reservations, project reviewing, order charging, bookings as well as *analytic* tasks such capturing meta-information about the samples as well as the generated raw as well as post-processed data and tracking the entire data processing workflow. At the Functional Genomics Center Zurich, we have developed a system called B-Fabric [27], which addresses all these requirements after having originally started back in 2005 in the same direction as the above-mentioned tools [26]. In the following, we will very roughly sketch the architecture of this system (see Figure 4) without going into further details that are beyond what is needed to understand the usage of such a system for instant data visualizations and exploration. In the B-Fabric system architecture, experimental data is produced by instruments and applications. This data typically resides on different *data storages*. The knowledge about this data, in particular about its location, is tracked in the *meta-database*. Instruments and applications use Web services to query and feed the *meta-database*. Thereby, all relevant structured data is linked together.

(1) Data production and generation is completely decoupled from the overall system. Data is produced, for instance, by exchangeable instruments, which usually come with their own storage. Data producers can easily be registered to the overall system. Instruments experts are working on this level to install and configure the instruments as needed, as well as to register them in the *meta-database* to couple them to the overall system.
(2) Data beamers, which are special applications running on the instrument’s storage, demultiplex and transfer the produced data into authorized storage. These data storages are strictly organized following commonly agreed rules. For instance, the data is stored in project-separated file folders, so-called containers, representing the access units for all data produced in a project. At this level, bio-informaticians, who are responsible for the maintenance of the different storages, are also responsible for implementing the data beamers based on their knowledge of the corresponding storages.
(3) The *meta-database* is the brain of the entire system [7] which holds all the knowledge relevant to the core facility. It documents the projects and orders of all users. It stores the samples processed in these projects and orders, as well as the relevant information about the applied procedures and workflows. Thanks to the applications, the *meta-database* also holds all necessary information about the produced data files and thus provide a homogenous overall picture over highly heterogeneous and autonomous *data storages*. Among other things, it allows for the questions of “Who created which data, in which project and with which samples and with which protocols?” An advanced role model implements a fine-grained access control such that the users can only see the piece of the data world that they are allowed to. The data of the *meta-database* is accessible via GUI interfaces as well as Web services. The entity concepts are realized in such a general manner that the *meta-database* is prepared to store any type of future research data.
(4) External applications exploit the meta data to access, process, and visualize the produced research data. The required data preprocessing, including the data crunching is usually performed on external infrastructures (clusters/grids/clouds, [38]). Visualization software, such as the shiny server [13], is an example of the implementation of an external application. The required data is fetched via the Web services of the underlying infrastructure. Web services can register the analysis result in the *meta-database* if needed.

Essential for this evolutionary architecture is implementing a highly generic application concept, which allows for implementing dynamic, ad-hoc workflows of any type to keep track of the entire data processing. Using preceding and succeeding relationships, all applications know on which data they can work [7].

The current B-Fabric 11 release is built upon Jakarta EE, JSF, PrimeFaces, OmniFaces (Web Application Development), PostgreSQL (Data Management), Apache Lucene (Fulltext Search), and SOAP (Web Service). B-Fabric has run in daily business at the Functional Genomics Center Zurich since February 2007. Table 2 lists some numbers showing its usage in our three technology platforms Genomics, Metabolomics, and Proteomics.

**Table 2:** Some numbers on system usage as of June 8, 2022.

Users	7411	Organizations	349	Samples	289,173
Projects	4285	Institutes	793	Data resources	2,021,621
Orders	24,030	Instruments	319	Workunits	228,013
Services	1866	Applications	259	Disk storage size	> 0.4PetaByte

## 3 Application

In this section, we exemplarily demonstrate how our platform is used for ad-hoc linking a visualization software solution to our system in a way that the entire user community can benefit from it. We use the R environment in the example below, but the interface described in this manuscript can be used by any other programming environment supporting Web Services Description Language (WSDL). While the first application example attaches a higher-level interactive user interface to the data management system, the second example is more of technical nature to demonstrate how the application programmer can glue together data, metadata, and visualization libraries on the R command line. Since we focus more on the technical description in the text, the caption will give more details on what is displayed on the visualization in Figures 6 and 7.

### 3.1 Setup and configuration

**Figure 5:**
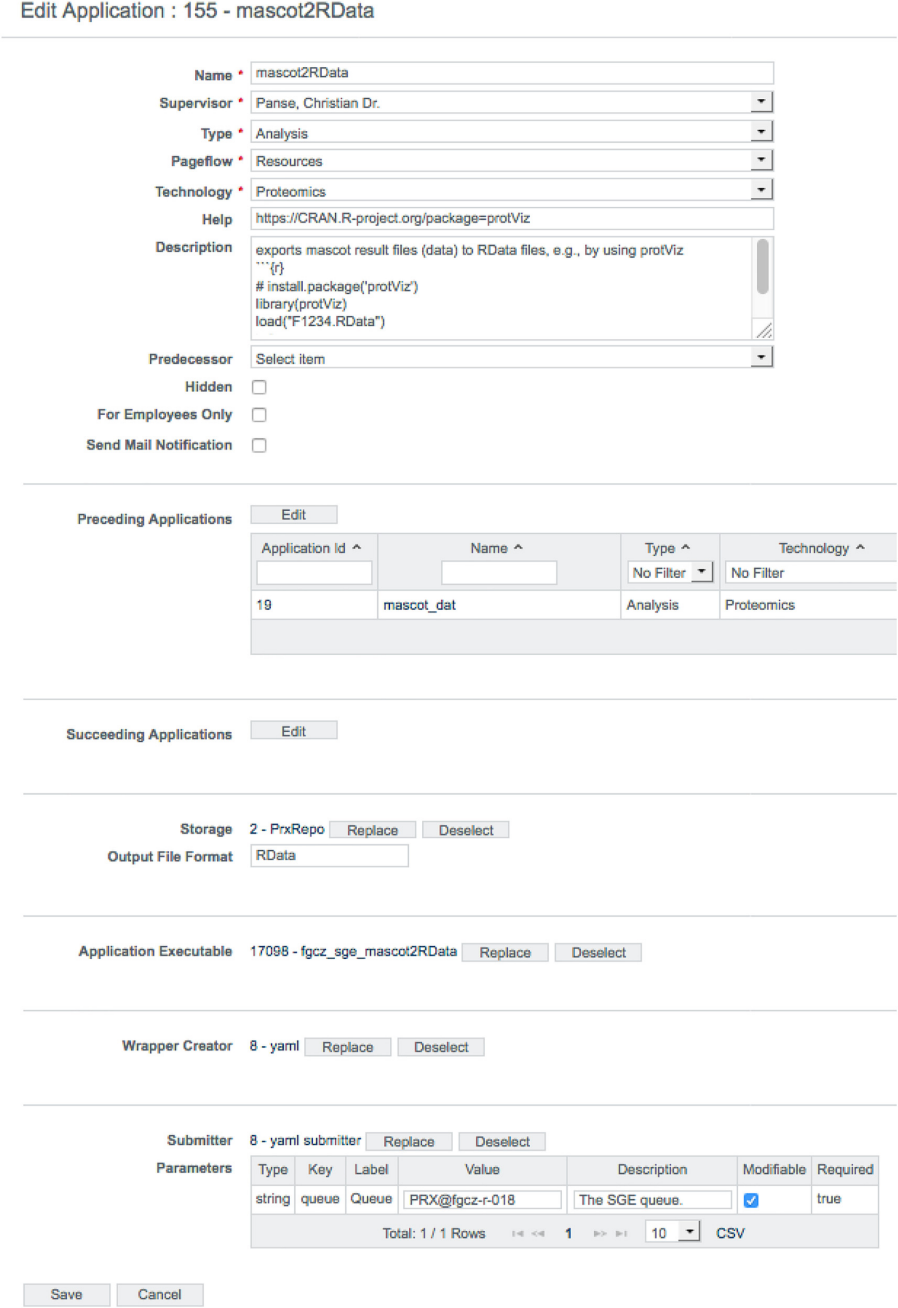
Application configuration screen of the B-Fabric platform.

Before an application can be applied to the data, it has to be configured properly. The B-Fabric platform not only allows to annotate metadata but also provides an annotation mechanism for maintaining the application configuration. As shown in Figure 5, B-Fabric allows registering applications with their specific properties, e.g., *type* or *technology*. The tremendous possible flexibility is achieved by attaching executables written externally in any language and allowing for wrapping this code by a previously registered wrapper creator, which provides the abstraction for feeding the application executable with the needed meta-data. In addition, the submitter abstraction allows running the application executable on a configured computation environment of your choice. Every application takes an input (in that context, it is also an application) and provides an output. The field *Preceding Applications* defines the application’s input, and ad-hoc workflows become a reality. The application ID=155 shown in Figure 5 requires as input a peptide identification search [14] application having an application ID = 19. Depending on the computational need, the jobs can be performed on a local compute-server or an attached cloud infrastructure (see [[38], section 4]). The wrapper creator mechanism implements the job staging. The XML listing below defines the environment where the application is executed and what script or binary file is used. It is also possible to define a list of arguments and pre-defined argument values to be passed to the application executable. The XML form for the executable configuration turned out to be easier to maintain and deploy, especially if the application requires a long list of user parameters.

**Listing 1**

Executable configuration as XML.

**Figure:**
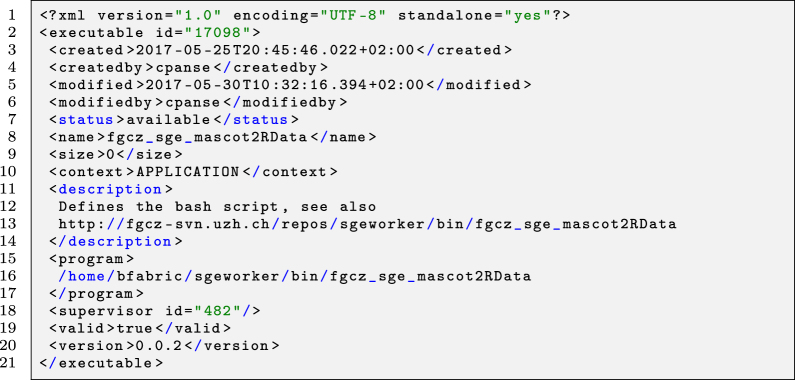


In the example using R shiny, all jobs created by that application (ID = 155) are available (given the user is authorized) in the platform’s user interface, e.g., by calling the module as shown in the following tiny code snippet.

**Listing 2**

Usage of the B-Fabric shiny module.

**Figure:**
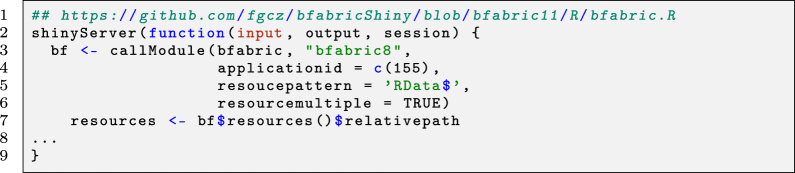


The retrieved metadata, e.g., sample information, storage, and the file path of each selected resource, are kept in the bf object (line 6). This object can later be used as a container for any programming purpose.

### 3.2 Interactive visualization

To provide a one-to-one example for the generic description of the platform sketched in Section 2 and Figure 4, we refer to each item number of that section.

(1) The recording of the raw data on a today’s mass spectrometer device can easily take up several days depending on the length of the liquid chromatography (LC-MS/MS) runs, the number of samples and the number of quality control (QC) runs scheduled by the systems expert. During data recording, the data is saved onto an instrument control computer. After completion, the data is automatically copied to a *data-storage* by robots which are responsible for the data transfer between instruments and data-storage.
(2) A local copy of the data will remain as a backup for two weeks on the instrument computer before deletion. A *data-storage* is typically a storage area network (SAN) and is attached to the data producing machines and the computing area through an optical fiber network. XFS turned out to be the file system of choice. Directly after synchronization of the data to the SAN (see arrow 2 in Figure 4) a first preprocessing will be triggered converting the proprietary raw file format into a generic machine-readable format, e.g., Mascot generic file (mgf) or mzXML. Ideally, the annotation (see arrow 1 in Figure 4) in the platform has already taken place prior to the data analysis but needs to be completed at last when the raw data is “imported” into *meta-database* where the corresponding raw data will be linked.
(3) As next step, the systems experts or scientists trigger a so-called “MS/MS database search”, e.g., by using Matrix Science Mascot Server [14] or comet [39]. Those search algorithms assign the mass spectrometric data (spectra) to protein sequences. The amino acid sequences were predicted by using DNA/RNA sequences. A quantitative measure is derived from the area under the curve (AUC) of the ion counts signals over the retention time. For further processing, this data is usually converted into an exchange format, usually XML (see also HUPO Proteomics Standards Initiative [40]). An XML conversion of such data has the drawback that the resulting files are very large, and the parsing is very time-consuming. As a practical step, the XML is further processed into a more typical R list, where if necessary, some filtering can already be applied and saved as compressed RData “container” [41]. Similar libraries and procedures are also available for the programming languages Python, Ruby, and Perl. After this step, the data can be read directly into the application.
(4) A system-specific R shiny module [42] is handling all the authentification and data staging tasks (see arrows 3 and 4 in Figure 4) using the Web services interface of the B-Fabric platform. The data staging is performed by using the network file system (NFS, RFC7530) or the secure shell (SSH, RFC4254) protocols. Also, for mobile computers, a proxy solution is possible.

**Figure 6:**
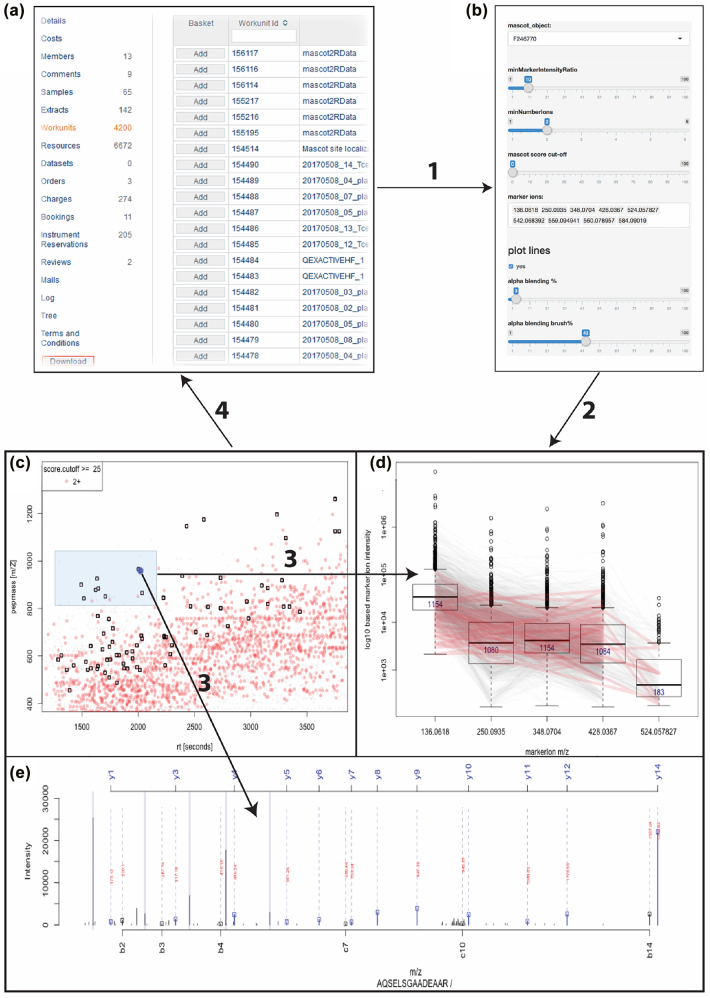
Example 1 – on using B-Fabric and shiny.

The screenshots in Figure 6 display the first application example with the B-Fabric platform (see also arrow 3 in Figure 4). The application software [14, 25, 28, 43] itself detects and quantifies known marker ions released during the different types of ion fragmentation of the peptide during the data acquisition. Together with the findings of the previously mentioned “MS/MS database search” and the dynamic graphical analysis, it is a powerful tool for the decision-making exploration process of the scientists. (a) The screenshot gives an overview of the research project statistics, e.g., the number of samples and measurements, by querying the *meta-database*. Particular resources can be collected into an “Amazon” basket and transferred as input to related applications. (b) The panel displays some relevant parameter settings for the selected application. Most important are the input containing the list of the mass pattern. (c) Each data item represents a picked peptide candidate signal on the m/Z versus retention time *zoomed-in* scatterplot of one LC-MS instrument run. Color represents the charge state (filtered; only 2+). The black boxes identify signals where the expected ADPr fragment ion mass pattern was detected using [25] package. (d) The boxplots display the distribution of the ion count of each tandem mass fragmentation ion pattern $mZ_{ADPr}=\left(136.0618,250.0935,348.0704,428.0367,524.057827\right)$ . The lines connecting each data item on the boxplot depict the trend. *Brushing*, the red colored lines selected by the blue box in (c), enable the “discovery mode” of the application. (e) Using *linking* the peptide-spectrum match is shown. The plot shows that the highest peaks are assigned to the b and y ions. Additionally, the marker ions are highlighted (Quality Control). The result of the exploration process and the used parameters can be made persistent by feeding it back to the *meta-database* and the *data-storage*.

### 3.3 Command line triggered visual exploration

The second application example illustrates the interaction between an application (performing quality control) and the B-Fabric platform on the command line level using the R environment. On the one hand, it shows how a more programming skilled user can use the platform interface. On the other hand, it also demonstrates how a more complex visualization application can be implemented. Of note, the workflow consists of steps **(3)** and **(4)** only (see Figure 4). The depicted code snippet in Listing 3 requires an authentification configuration, saved in login and webservicepassword in line 11, which is used for accessing the B-Fabric platform.

The example screens a smaller dataset generated as part of an experiment to identify the entire fruit fly model organism [3]. Lines 11–13 perform a database query via WSDL to the B-Fabric platform. This particular query returns all resources belonging to a given workunitid=165473. A workunit is a collection of files. For a more complex example like an *expression analysis*, we could also retrieve additional metadata, e.g., the sample annotation and treatment for each measurement. Besides the read mechanism, the webservice methods save, and delete can be used to save or delete the result of an analysis. By using the save method also, intermediate result files can be uploaded to B-Fabric. Lines 15–16 concatenate the root directory with each resource’s extracted relative file path. For our example application, to read the content of the proprietary instrument files and visualize it, we use an R package rawDiag [44], a software tool supporting rational method optimization by providing MS operator-tailored diagnostic plots of scan level metadata. Lines 19–21 read the proprietary mass spectrometry formatted data in parallel. This method can provide the mechanism of retrieving the data via remote method invocation (RMI) or using the secure shell protocol (SSH). The idea is to avoid copying the entire file.

**Listing 3**

**Figure 7:**
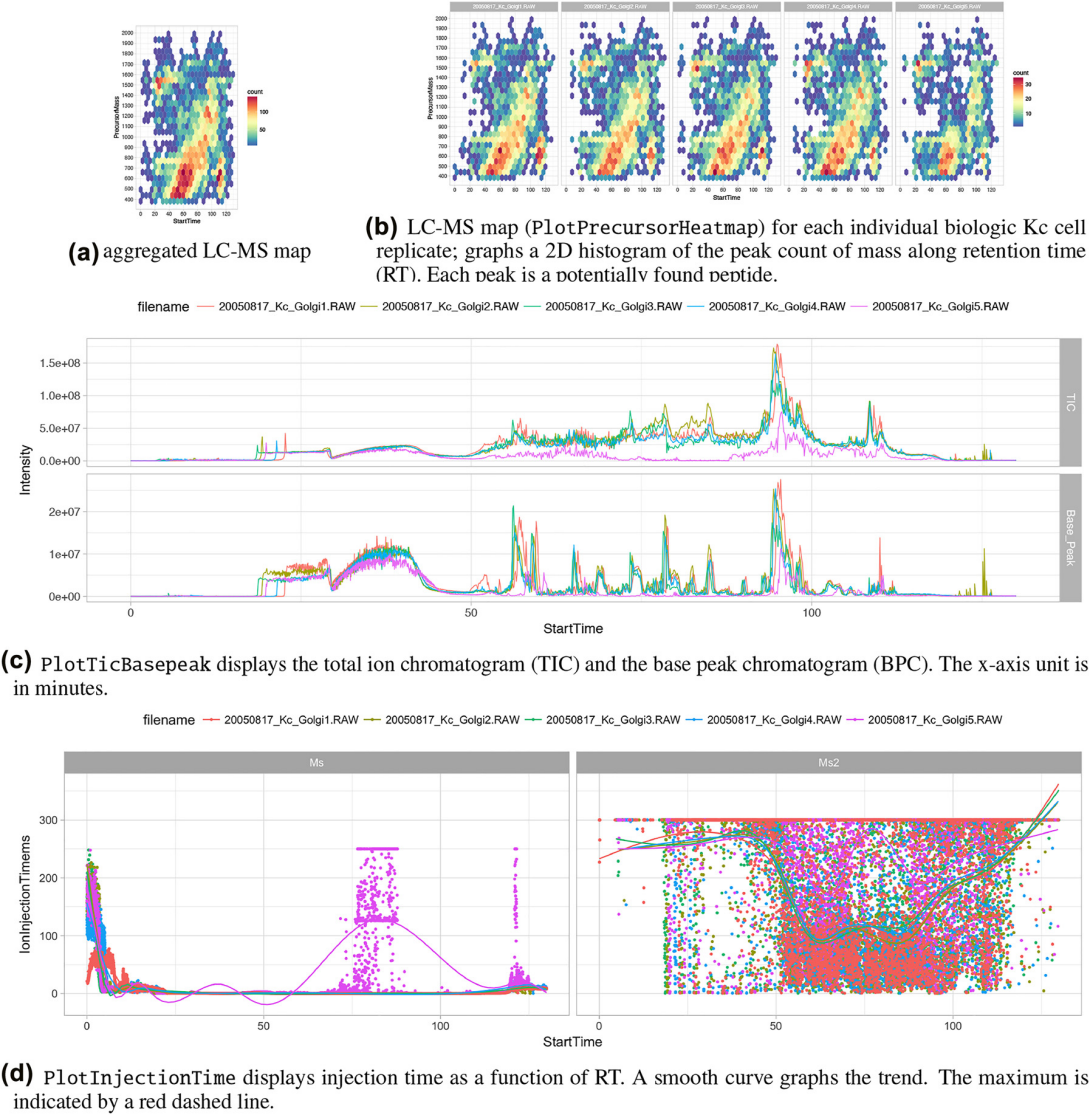
Example 2 – visual exploration using the R command line.

R command line code snippet producing the results in Figure 7.

**Figure:**
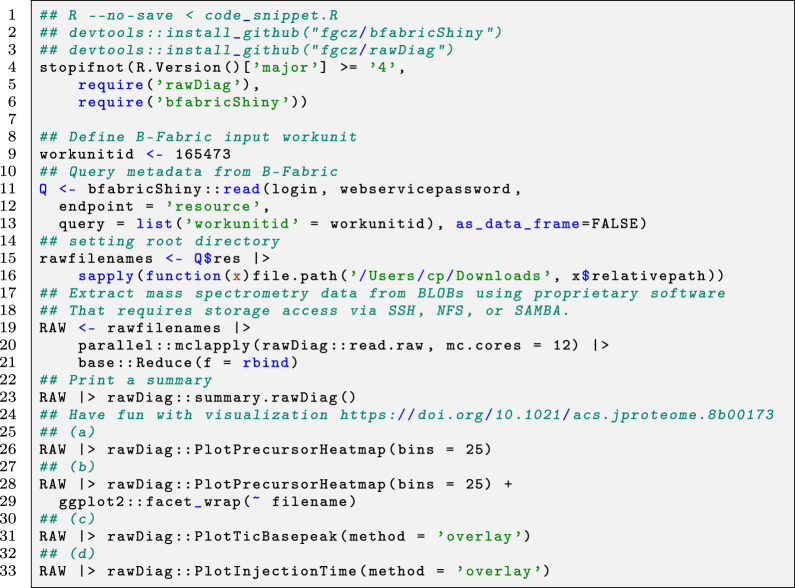


From line 24 on, we generate the graphics displayed in Figure 7. The graphics on Figure 7 (b) can also be used to perform visual representation as a set of thumbnails of a bigger data set [45]. The design of generating the visualizations follows the concept described in [21] using [46]. The rawDiag package [44] can be used stand-alone or plugged into the B-Fabric system http://fgcz-ms-shiny.uzh.ch:8080/bfabric_rawDiag/ by using the bfabricShiny module [42]. It is used as an application running in a web browser and from the R [9] command line.

Furthermore, as input for Figure 7, we use data measured on a Thermo Finnigan LTQ device (2005) using 96 min LC gradient. Injected were typically digested Golgi Kc cell line replicates derived from a fruit fly (*D. melanogaster*) [3], see also Figure 1(a). Figure 7(a) graphs an aggregated “overview map” of the liquid chromatographic-mass spectrometry (LC-MS) runs of the five replicates. The 2D map shows a typical pattern. Which means. After a loading phase (15 min), lighter peptides start to elute, followed by heavier ones. Figure 7(b) graphs individual LC-MS runs. Replicate five, 20050817_Kc_Golgi5, shows a different pattern after 50 min. Figure 7(c) Also, the total ion count (TIC) drops after 50 min (pink curve). In Figure 7(d), the injection time plot graphs the time used for filling the ion trap of the mass spec device for the scans of MS level 1 (left) and level (2) right. At the latest, seeing the point cloud after 60 min on the graph (d) indicates a malfunction often caused by the spray attached to the ion source.

## 4 Lessons learned

Deploying the newest visualization and exploration solutions to a productive environment is challenging. The described solution based on using the B-Fabric platform enables the linking of various visualization, data processing, and data analysis software tools to a data management system containing massive repositories of Life Sciences data and metadata, such as definitions of applied experimental designs. The platform analyzes massive amounts of genomics, sequencing, proteomics, and metabolomics data generated at our core facility for over a decade. Over the years, we witnessed the rapid evolution of technologies in the various Life Sciences areas, which consequently demands an integrative platform ready to couple with any potential future application with little effort. Concretely, we learned the following lessons:

*Every institution has its superior solution.* Concerning core facilities, the systems are often adapted to the internal business needs, for instance, reviewing, accounting, ordering, and all other required administrative processes. Other facilities usually rely on commercial lab information management systems (LIMS). Once a LIMS is in operation, it is almost impossible to exchange such a system due to the enormous financial and human capital investment. Usually, LIMS are implemented for a specific domain and are not generic enough. From the software engineering point of view, it is essential to have a generic platform providing an interface to read and save *any* type of data.

*Follow commonly defined standards.* Sticking to standards in packaging analysis software eases the deployment and teaching of the software packages. Applications for statistical modeling, differential expression analysis, p-value adaption, data mining, and machine learning, e.g., data imputation missing values and information visualization techniques. Platforms such as the Bioconductor [47, 48] already provide solutions for more than a decade, but the deployment and the provisioning of data and metadata (containing the experimental design) to the scientist having the domain knowledge remain unsolved.

*Divide-and-conquer.* In an early phase, more than ten years ago, our system was designed as one monolithic stand-alone system where all applications were integrated into a platform with one programming language. As a result, the software engineering tasks were distributed over a few shoulders (software engineers). Consequently, releasing small changes in an analysis module took too long. The *decoupling* of the data management and the application part led to decoupling and fully parallelization of the development cycles. Software engineering groups and statisticians/bioinformatics groups develop with different release times, using different development tools and programs in different languages. As an additional benefit of the separation, the analysis tools can also be deployed on a different site running on different systems, which leads to more robustness of the code, allows better reproducibility, and finally enables more transparency to the R research. Good examples of pluggable visualization are shown in Section 3 [25, 44]; others are examples are described in [11, 49, 50].

**Figure 8:**
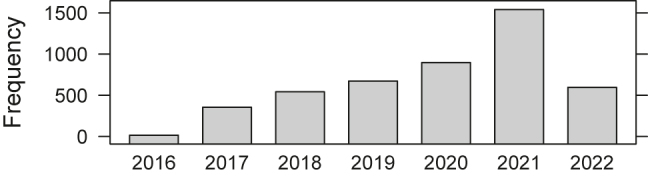
Usage frequency ∼ year (May 2022).

Since we introduced the described concept in 2016, we have a significant increment in the number of executed applications. Note, in the graphic, we only counted if the result of an application is stored (see Figure 8).

*Eliminate time-consuming user interaction by increasing user satisfaction.* Detect and fix issues before a large group of users recognizes them and creates several tickets for the same issue. Thereby, you increase user satisfaction and avoid time-consuming communication. Also, a common, intuitive interface saves time for training and code adaption 

*Sustainability through consistent metadata annotation.* An essential requirement for effective data analysis is a high-quality metadata annotation encoding the experimental design. Even so when, as in our case, a type of most project duration is between two and five years (as shown in Figure 2). For instance, the best visualization tool/technique is worthless if the visualized data cannot be linked to the corresponding metadata, which builds the fundament for the correct interpretation of the visualized data.

## 5 Conclusions

In this paper, we have sketched how ad-hoc visualizations and analytics can be realized on top of an integrative platform that captures and processes any kind of data and scientific processes. For that, we were using our integrative B-Fabric platform. The key idea behind the platform is to decouple the metadata management from the data processing and visualization components. The advantage is that the developments in the different areas can take place at different speed, and each area can benefit from using specific software environments, which provide the basis for realizing optimal solutions in the corresponding areas. The two chosen implemented application examples illustrated how the visualization tools could interface in such a platform. The evolutionary B-Fabric platform has already proven its capability in daily business for more than a decade, especially by providing interactive visual data exploration solutions in the omics areas to a broader class of users. The platform enables ad-hoc attaching visualization tools and makes them available to a vast set of annotated data and an entire user community. Since parameter and intermediate results can be instantly made persistent, the platform is also a milestone towards the trend of *reproducible research*. It provides the fundament of a maintainable and robust software stack that is not limited only to the area of Life Sciences . Furthermore, in this way, B-Fabric helps scientists to meet requirements from funding agencies, journals, and academic institutions to publish data according to the FAIR (Findable, Accessible, Interoperable and Reusable) [51] data principles.

Availability: All R packages [44, 49, 52, 53], including documentation, unit tests and examples, are available under the GNU General Public License v3.0 via the following URLs: https://github.com/fgcz/rawDiag, https://bioconductor.org/packages/rawrr/, https://bioconductor.org/packages/NestLink/, https://github.com/fgcz/bfabricShiny, https://CRAN.R-project.org/package=protViz. The B-Fabric data management platform is available for all registered users of the Functional Genomics Center Zurich through https://fgcz-bfabric.uzh.ch. The code of B-Fabric is closed since it is commercially licensed. Therefore, we restricted the presentation of the platform to a rough level that is needed to understand how omics workflows can be realized with an integrative platform such as B-Fabric. Interested parties are welcome to approach Can Türker.
